# Implementation of Audio-Computer Assisted Self-Interview (ACASI) among adolescent girls in humanitarian settings: feasibility, acceptability, and lessons learned

**DOI:** 10.1186/s13031-016-0098-1

**Published:** 2017-01-04

**Authors:** Kathryn Falb, Sophie Tanner, Khudejha Asghar, Samir Souidi, Stan Mierzwa, Asham Assazenew, Theresita Bakomere, Pamela Mallinga, Katie Robinette, Woinishet Tibebu, Lindsay Stark

**Affiliations:** 1 International Rescue Committee, 122 E 42nd St, New York City, NY 10168 USA; 2 International Rescue Committee, 3 Bloomsbury Place, London, WC1A 2QL UK; 3 Columbia University Mailman School of Public Health, 60 Haven Avenue, B-4, Suite 432, New York, NY 10032 USA; 4 Population Council, One Dag Hammarskjold Plaza, New York, NY 10017 USA; 5 International Rescue Committee, TK International Bldg. 6th Floor, Bole Rd, Addis Ababa, Ethiopia; 6 International Rescue Committee, Bukavu, South-Kivu Democratic Republic of the Congo

**Keywords:** ACASI, Tablets, Technology, Humanitarian, Ethiopia, DRC, Research methods, Self-interview, Girls, Gender

## Abstract

**Background:**

Audio-Computer Assisted Self- Interview (ACASI) is a method of data collection in which participants listen to pre-recorded questions through headphones and respond to questions by selecting their answers on a touch screen or keypad, and is seen as advantageous for gathering data on sensitive topics such as experiences of violence. This paper seeks to explore the feasibility and acceptability of using ACASI with adolescent girls and to document the implementation of such an approach in two humanitarian settings: conflict-affected communities in eastern Democratic Republic of Congo (DRC) and refugee camps along the Sudan-Ethiopia border.

**Methods:**

This paper evaluates the feasibility and acceptability of implementing ACASI, based on the experiences of using this tool in baseline data collections for COMPASS (Creating Opportunities through Mentorship, Parental involvement, and Safe Spaces) impact evaluations in DRC (*N* = 868) and Ethiopia (*N* = 919) among adolescent girls. Descriptive statistics and logistic regression models were generated to examine associations between understanding of the survey and selected demographics in both countries.

**Results:**

Overall, nearly 90 % of girls in the DRC felt that the questions were easy to understand as compared to approximately 75 % in Ethiopia. Level of education, but not age, was associated with understanding of the survey in both countries.

**Conclusions:**

Financial and time investment to ready ACASI was substantial in order to properly contextualize the approach to these specific humanitarian settings, including piloting of images, language assessments, and checking both written translations and corresponding verbal recordings. Despite challenges, we conclude that ACASI proved feasible and acceptable to participants and to data collection teams in two diverse humanitarian settings.

**Electronic supplementary material:**

The online version of this article (doi:10.1186/s13031-016-0098-1) contains supplementary material, which is available to authorized users.

## Background

Audio-Computer Assisted Self- Interview (ACASI) is a method of data collection in which participants listen to pre-recorded questions through headphones and respond to questions by selecting their answers on a touch screen or keypad [1]. ACASI is believed to improve quality data collection, above and beyond general benefits of technological approaches to data collection which can minimize data entry errors and reduce time to clean and monitor incoming data [2]. Such benefits of using ACASI may include minimizing skipping of questions and increasing reporting of sensitive behaviors [3–6]. The use of ACASI among adolescents in low- and middle-income countries has been growing in recent years. Topics have typically centered on sexual health, drug use, sexual risk-taking, or other sensitive behaviors [4, 7] due to ACASI’s potential to reduce the social desirability bias that may occur in face-to-face interviews [8]. However, some criticisms of ACASI suggest the administration format may eliminate the empathy, trust and rapport that face-to-face interviews provide, which may provide other opportunities for disclosure [9–12] or outreach for referral services. The appropriateness of technological approaches like ACASI to elicit sensitive information like sexual behavior from very young adolescents has also been noted [13]. While ACASI is increasing in popularity as a data collection method in development settings, little is known about its use in humanitarian emergencies. In particular, settings with refugees and/or internally displaced persons can be challenging environments for survey administration given levels of mobility and the fact that large, heterogeneous groups of people with various cultures and languages which often reside in close geographic proximity. Such heterogeneity may require surveys to be administered in multiple languages or require cultural adaptations that may increase the potential for measurement error or require substantial updating of the tools in the field. Extensive efforts for rigorous electronic survey administration of sensitive information may be difficult in such settings. In addition, there may also be challenges directly related to the use of ACASI, including concerns regarding risk of theft of portable technological devices [14] against the backdrop of limited or interrupted internet connectivity and electricity.

Thus, the current paper seeks to explore the feasibility and acceptability of using ACASI with adolescent girls and to document the implementation of such an approach in two humanitarian settings: conflict-affected communities in eastern Democratic Republic of Congo (DRC) and refugee camps along the Sudan-Ethiopia border. Specifically, this paper draws from experiences related to the baseline data collection for the COMPASS (Creating Opportunities through Mentorship, Parental involvement, and Safe Spaces) impact evaluations in DRC and Ethiopia. COMPASS is a comprehensive program that delivers a curriculum for adolescent girls in safe spaces and engages community members, service providers, and caregivers to create a supportive environment for girls to protect them from violence and encourage a healthy transition to adulthood. Primary outcomes of the COMPASS study include changes in sexual violence, unhealthy personal relationships, and early/forced marriage, among other gender-related outcomes. The primary objective of the present manuscript is to outline the preparation process for using ACASI in these settings, the technological specifications of the tools, and lessons from its implementation.

## Methods

### Study design

COMPASS, a multi-country research study, is led by Columbia University, in partnership with the implementing organization, the International Rescue Committee (IRC). In DRC, the baseline for the cluster-randomized controlled wait-list trial was implemented between May-July 2015. The overarching objective of this evaluation is to understand the incremental effectiveness of the parental engagement activities in addition to the core COMPASS components on girls’ experiences of violence and well-being compared to girls whose parents have not yet received the parental activities. In Ethiopia, the baseline for the cluster-randomized controlled trial was implemented between August-September 2015, which seeks to understand the overall impact of the COMPASS program on girls’ experiences of violence and well-being compared to the wait-list group. Girls aged 10–14 years in DRC and 13–19 years in refugee camps in Ethiopia were asked to participate in the study and complete a baseline survey. In total, 869 girls from DRC and 919 girls from Ethiopia completed the survey. All girls that had enrolled in the program were eligible for the study, and girls were formed into program groups based on geographic proximity and language. Groups were then randomly assigned to either the intervention group or the control group. Additional study details are found elsewhere [15].

### Preparation of ACASI

The following subsections document the process of developing and using the ACASI technology, with particular focus on language and translation, use of visuals, and technical specifications.

#### Languages & translation

The original intention behind selecting ACASI was to improve the quality of reporting of sensitive experiences, such as forced sex and sexual history, which in Ethiopia was asked to all girls (13–19 years) and in the DRC was asked only to older girls in the study population between 13–14 years of age. In DRC, ACASI was selected to complement the CAPI (Computer-Assisted Personal Interview) sections of the survey which included less sensitive questions around demographics or gender norms, for example, and was administered by trained female data collection staff. Interview tools were translated and back-translated into Mashi and Swahili, the languages of survey administration. Final translations were checked with groups of girls to ensure the language was comprehensible for the target groups. Following this final check, audio files of questions and text were programmed into the tablets. Changes were made as needed during the baseline training.

In refugee camps in Ethiopia, the survey was originally to be implemented in Arabic, which is the language generally spoken in markets or community spaces in the refugee camps. However, formative assessments with girls revealed that understanding was not sufficient for survey implementation. Subsequently, a language assessment was conducted by IRC’s research and program teams which identified 19 tribal languages in the camps. Four of the most prevalent languages were selected for the survey to meet sample size requirements: Meban, Regarig, Ingessena Kulelek and Funj/Berta, which are all non-written languages spoken in Sudan. It was decided that the research would be conducted solely using ACASI, because the languages were non-written and low literacy levels made it unlikely that simultaneous translation by female enumerators from English or Arabic into a local language would be feasible. Refugee women were hired as ACASI assistants to set girls up with the tablets and demonstrate how to use it as they were able to communicate with girls in the local languages.

Translation therefore followed a careful process with a large team: male IRC community workers with adequate levels of literacy verbally translated the questions from English to the target language, and discussed the meaning and wording with a group of 2–3 non-literate refugee women and a native English speaker. One woman then audio recorded the question from memory after phrasing was agreed upon. This was then used as the basis for back-translation and reconciliation of any discrepancies between the original English questions. Final recordings were checked with girls to ensure they had a strong understanding of the questions. Once audio files were programmed into the tablets, the recordings were checked and amended a final time as needed during baseline training and piloting.

#### Response categories & images

**Fig. 1:**
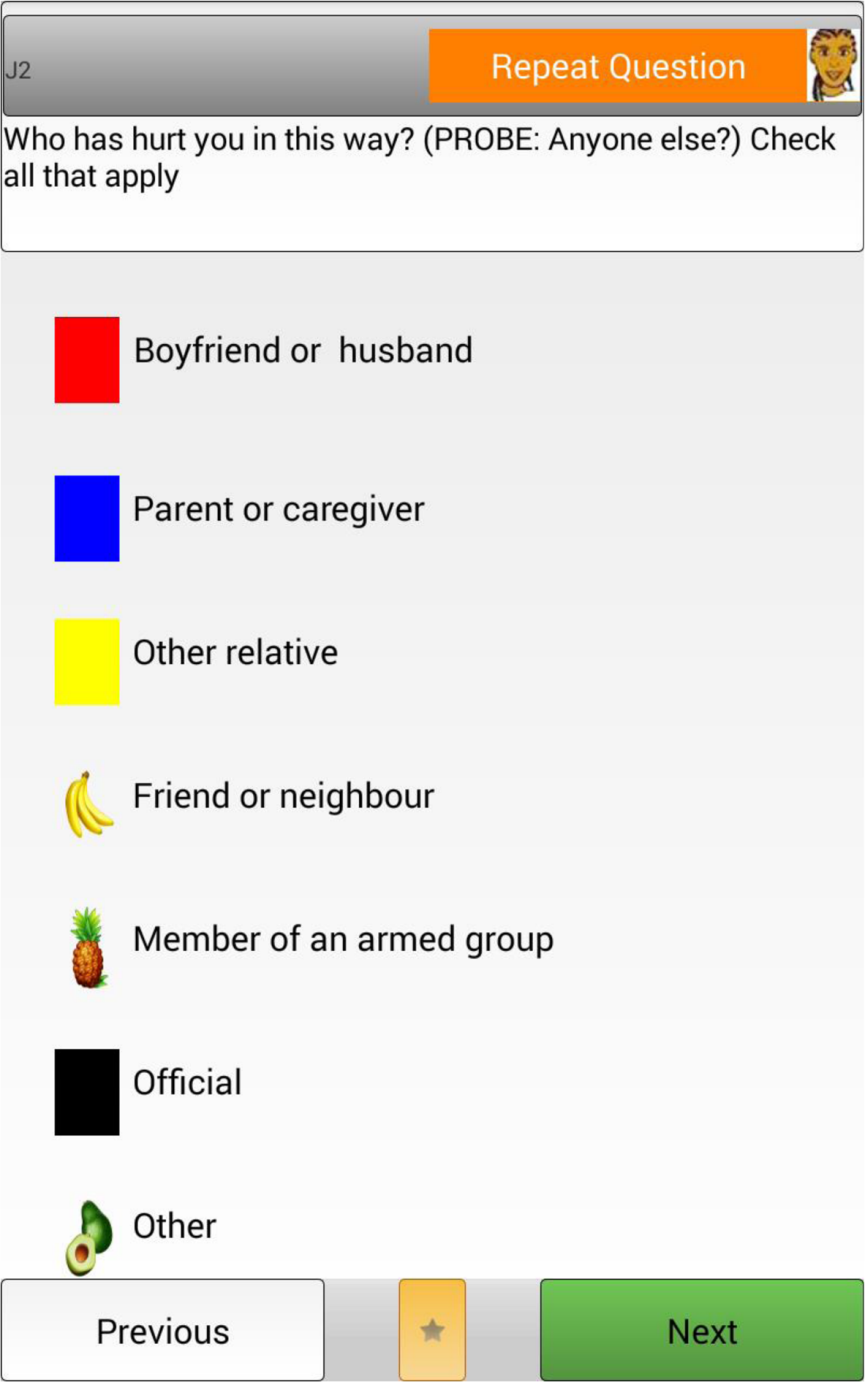
Example interface of ACASI screen for girls’ questionnaire

The ACASI screen generally displays the written question at the top of the screen, including a button with a graphic to repeat the audio, with the options listed below next to colored buttons or images (see Fig. 1). A maximum of eight options is recommended for ACASI, plus colors for ‘don’t know,’ ‘previous question’ and ‘next question’ buttons.

Given contextual differences in colors or potential images, ACASI response option colors and images were piloted with girls in DRC and Ethiopia. In DRC, only six colors could be consistently named by all girls from these communities when tested which would have limited the potential response options in the ACASI tablet. Girls were not able to differentiate between shades of a single color and translation challenges made it difficult to describe shades of color for ACASI response options. Girls who had not been to school found recognizing colors particularly challenging; common fruits and vegetables were easier for them, though caution had to be exercised in the selection of images (there are multiple words for different types of banana in some languages, for example). While ACASI options had been programmed in local languages, it was found that individuals who had been to school were mainly familiar with the words for various colors in French (as taught in school) and not in the local language they spoke. Girls were given a sheet during survey administration that showed the response options and the corresponding words in their local languages. Testing found that a mixture of identifiable fruits and colors was the most appropriate way to have eight recognizable and distinct options for the target group in DRC (see Fig. 1). Previous research has documented that although some specifications of graphics or images (e.g., size; brightness of color) may influence study responses, they have not been fully examined in technological approaches to data collection, such as ACASI or CAPI [16]. In Ethiopia, it was found the ten colors could be used in the ACASI screen for responses; thus no images such as fruits or vegetables were needed for response categories.

#### Technological specifications

The present study used Android-based tablets which were programmed in New York City by the Population Council. The surveys on the tablets were updated as needed when wireless internet was available in country. Data from tablets was uploaded to a laptop computer in each country and exported to the research team via a secure cloud-based system. Further technological specifications are found in the Additional file 1.

#### Training data collection staff for administering ACASI

In Ethiopia, most of the ACASI assistants supporting the girls had very low levels of literacy and were also new to touch-screen technology; training was therefore extremely important in preparing them for data collection. Program staff assisted potential ACASI assistants with low English literacy in writing resumes and cover letters that could be submitted as an application of interest. Training occurred in phases: a basic introduction to handling the tablets progressed to practice with listening to the audio and selecting relevant responses, and then to interview simulations where ACASI assistants explained tablet use to each other, using the audio as a reference for practice question content. Lastly, the team conducted a pilot with 10 volunteers from one of the camps. Translators in the 4 languages used during data collection were present during training, and refresher trainings were conducted prior to launching data collection at subsequent camps after the first camp’s data collection was completed. Using young women as data collection staff from target communities proved valuable; their similarity to the girls in terms of background and age were important for their ability to guide the girls through the survey process. The training in DRC followed a similar pattern and piloting of tablets with girls and caregivers in communities. In terms of data collection staff themselves, skills were assessed in the initial training to gauge their comfort and understanding of ACASI in both countries. In Ethiopia, ACASI assistants who had very low levels of literacy were matched with ACASI assistants who had a better understanding of tablets from other camps for additional support throughout data collection. DRC data collectors were literate in French and either Mashi and/or Swahili (languages of survey administration).

### Measures

At the conclusion of the survey, one question was asked to assess the acceptability and perceptions of the survey and using ACASI: “Do you feel the questions were easy to understand?”. Responses were based on a Likert scale from *very easy to understand* to *very difficult to understand*. A subsequent fill-in question posed to the participants was completed by the data collection staff: “What did you think of using the tablet?” in DRC only. This was asked only in DRC given the non-written nature of languages, low literacy levels of ACASI assistants in written languages such as English or Arabic, and limited availability of literate staff to write in responses while overseeing the data collection in the refugee camps in Ethiopia.

### Analysis

Descriptive frequencies were tabulated separately based on selected demographics for each country for the quantitative question using STATA. Wald chi-square tests were used to assess statistically significant differences in responses by demographics. Where appropriate, odds ratios were constructed in logistic regression models between demographics and responses related to survey understanding. In regards to the fill-in question, results were categorized into the following: (1) fine, comfortable happy; (2) very good; (3) enjoyed using the tablet;(4) very easy; (5) a little difficult; (6) very difficult; (7) fear; (8) tired; or (9) response unclear.

## Results

Response rates for the survey was high in Ethiopia and DRC (97.8 % and 99.8 %, respectively). Overall, nearly 90 % of girls in the DRC felt that the questions were easy to understand as compared to approximately 75 % in Ethiopia (see Table 1). Amongst girls in the DRC sample, girls were classified as older at ages 13 or 14, and younger at ages 10–12 (whom only completed the CAPI version of the survey). There were no significant differences in understanding of questions by age group. There were significant differences in understanding of questions by school attendance in DRC. Girls who had ever attended school were 1.76 times more likely to say that the questions were very easy to understand, as compared to girls who had never attended school (1.14–2.71, *p* = 0.01). There were significant differences in question comprehension by educational attainment. Girls who had completed some or all of secondary school were 7.91 times more likely to report that the questions were very easy to understand than girls who had completed some or all primary school (1.08–58.00, *p* = 0.04). In Ethiopia, girls were classified as ‘older’ at ages 16–19, and ‘younger’ at ages 13–15. Girls who had ever attended school were 1.80 times more likely to says that the questions were easy to understand, as compared to girls who had never attended school (1.29–2.51, *p* = 0.001). Differences in question comprehension were not significant by age group (*p* = 0.33) or educational attainment in Sudan/South Sudan (*p* = 0.53) or Ethiopia (*p* = 0.20), though access to education was low across the board.

**Table 1:** Frequencies and unadjusted odds ratios of demographics and understanding of survey

	Overall		Age						Schooling																	
	*N* = 868		10-12 (*N* = 491)		13-14 (*N* = 377)				Have ever attended school (*N* = 691)		Have never attended school (*N* = 178)				Completed primary as highest level of education (*N* = 637)		Completed secondary as highest level of education (*N* = 53)									
	(*n*)	(%)	(*n*)	(%)	(*n*)	(%)	OR	*p*-value	(*n*)	(%)	(*n*)	(%)	OR	*p*-value	(*n*)	(%)	(*n*)	(%)	OR	*p*-value						
DRC																										
Yes	747	86.06	413	84.11	334	88.59	-	ns	605	87.55	142	80.23	1.76	0.01	552	86.66	52	98.11	7.91	0.04						
No	120	13.82	77	15.68	43	11.41			85	12.3	35	19.77			84	13.19	1	1.89								
Ethiopia																										
	*N* = 917		13-15 (*N* = 704)		16-19 (N = 205)				Have ever attended school (*N* = 636)		Have never attended school (*N* = 252)				Completed primary as highest level of education in Sudan/South Sudan (*N* = 615)		Completed secondary as highest level of education in Sudan/South Sudan (*N* = 9)				Completed primary as highest level of education in Ethiopia (*N* = 615)		Completed secondary as highest level of education in Ethiopia (*N* = 7)			
	(*n*)	(%)	(*n*)	(%)	(*n*)	(%)	OR	*p*-value	(*n*)	(%)	(*n*)	(%)	OR	*p*-value	(*n*)	(%)	(*n*)	(%)	OR	*p*-value	(*n*)	(%)	(*n*)	(%)	OR	*p*-value
Yes	647	70.56	497	69.9	150	72.82	-	ns	473	74.37	157	62.3	1.8	0.001	455	73.98	7	77.78	-	ns	456	74.15	6	85.71	-	ns
No	215	23.45	172	24.19	43	20.87			129	20.28	77	30.56			127	20.65	1	11.11			128	20.81	0	0		

In terms of fill-in responses regarding what girls thought of using the tablet, an overwhelming 97 % of girls in DRC had a positive reaction. Nearly 65 % of responses were categorized as fine, comfortable, or happy; 19 % of girls reported that the tablet was very easy; and 7 % and 5 %, respectively, found the tablet very good or enjoyed using the tablet. Three percent of girls found the tablet a little difficult or very difficult to use, with a few reporting that they were a little afraid of it. No fill in responses were included in the Ethiopian survey given the non-written nature of languages.

## Discussion

The use of electronic data collection via ACASI systems to collect data from young adolescent girls on sensitive behaviors such as experiences of violence and sexual history is a new phenomenon in humanitarian settings. Findings of the baseline data collection for the COMPASS trials demonstrate that while overall understanding of the survey was acceptable, girls who had more education demonstrated higher likelihood of understanding. Additional time may be needed for researchers to give instructions on how to use the tablets to girls with lower educational attainment. Concerns of the appropriateness of ‘high-tech’ strategies have been noted elsewhere for data collection with younger adolescents [13]. Our results show that these strategies are feasible and understanding does not vary by age, but rather by educational level. Additionally, other forms of data collection such as engaging or culturally specific and empathic face-to-face quantitative interviewing techniques have also been used for adolescents regarding sensitive behaviors [10]. However, given the vast heterogeneity of ethnicities and languages found in our study sample, particularly among refugee camps in Ethiopia, this technological approach aided in survey administration. The use of ACASI also ensured anonymity and confidentiality of girls’ reported experiences in violence. Thus, all study respondents received a debrief script and referrals after survey administration, regardless of whether girls reported violence or not.

Additional lessons learned from implementing ACASI technologies are described below.

### Logistical considerations

Scoping of wireless connectivity and electricity availability in each setting was helpful to adjust study implementation scheduling as needed to ensure adequate updating of tools and transfer of data as needed. The tablets had sufficient battery to last a full day, and teams were generally able to charge overnight, or rely on back-up tablets. However, in DRC, some teams experienced difficulty in finding a reliable source of electricity and were therefore unable to charge tablets in full in order to complete all scheduled interviews during the following day. Each team had one back up tablet and at times a nearby team was able to send over their back up tablet in a study designated vehicle to assist with completion of interviews. Interview schedules had to be adapted to account for partially charged tablets.

### Risk of theft

The use of tablets in communities with little exposure to computerized technology drew attention from other community members, increasing the vulnerability of theft. During data collection in the DRC, five tablets stored in one bag were stolen out of a study vehicle. The team had backed-up data to a master tablet on a daily basis, but unfortunately this tablet was stolen too, resulting in the loss of 19 completed (de-identified) surveys. Lessons learned suggest that if data is backed up during the week on a different master tablet that such tablets are kept in separate areas.

### Observations from data collection staff

When demonstrating the use of tablets and ACASI to girls, some survey staff reported that a small number of girls were worried about touching the screen because they were concerned they might break it and would be responsible for replacing the tablet in DRC. Others tried to flip the screen like pages of a book or responded to questions by saying their answer out loud rather than touching a screen. Survey administrators remained near the girl while maintaining her privacy so that they could quickly respond to concerns or questions. In DRC, some supervisors and data collectors began to do individual and group introduction sessions to the tablet to address any concerns before beginning the survey. Allowing girls to simply touch the tablet and headphones and see other girls doing the survey improved comfort with the tablets. Headphones for ACASI were also cleaned with a wipe in front of girls before asking them to put on the ear pieces. In Ethiopia, the ACASI assistants remained near the girls who were completing the survey to assist in any questions.

### Limitations

Interpretations of the process of implementing ACASI and lessons learned should be considered with limitations in mind. First, a comparison between the use of ACASI versus other forms of interviewing such as face-to-face paper and pencil surveys would have been useful to discern differences in reporting of sensitive violence experiences. In addition, further qualitative data collection after the interviews were completed to gain further insight into the acceptability of ACASI would strengthen the lessons learned. There is limited published research available regarding violence experiences among adolescent girls in humanitarian settings; thus, comparison of perceptions of the feasibility and acceptability of ACASI and tablet-administration is limited.

In addition, a small number of girls sought referrals following interviews. One potential limitation, although untested, is that this number of girls seeking referrals could be lower if compared to a situation where trust and rapport was built with an in-person interviewer. Additional data to examine the potential differences between interview formats in terms of referral patterns or other potential differences in psychosocial wellbeing following such a sensitive interview would be useful to include in future research.

## Conclusion

Based on these baseline data collection experiences, ACASI proved feasible and acceptable to participants and to data collection teams in two diverse humanitarian settings. Admittedly, financial and time investments to ready ACASI were substantial in order to properly contextualize the approach through piloting of images, language assessments, and checking both written translations and corresponding verbal recordings. In refugee camps in Ethiopia, it was particularly useful given the numerous non-written languages of the study population. Despite these challenges, ACASI was successfully implemented in order to collect sensitive data among adolescent girls as part of the COMPASS trials.

## Additional file

Additional file 1: Technological Specifications. (DOCX 13 kb)
