# Supplementary material for: Implementation of Audio-Computer Assisted Self-Interview (ACASI) among adolescent girls in humanitarian settings: feasibility, acceptability, and lessons learned
Source: Confl Health. 2017 Jan 4;10:32. doi: 10.1186/s13031-016-0098-1 (PMC5209867; doi:10.1186/s13031-016-0098-1)
Supplement: Additional file 1: — Technological Specifications. (DOCX 13 kb) [file 13031_2016_98_MOESM1_ESM.docx]

# Technological Specifications

## Base tool for use to provide ACASI and CAPI

**Due to the** global nature of this study and its partners, Android-based tablets were purchased in the USA and programmed in New York by the Population Council. Original programming occurred in the USA as there were concerns about downloading software and substantial remote updates on slow and unreliable internet connections in the DRC and Ethiopia. This, in turn, could increase the chance of problems or malfunctions with the system. Final checks were carried out once the tablets arrived in country. The core ACASI/CAPI user-facing front-end is written in Java for Android using the open-source SQLite database to provide the database engine that contains the survey questions, flow logic, translations, question type parameters and pointers to audio recordings. This was programmed using the open-source Eclipse development environment.

## Updating and troubleshooting tablets in the field

The data collection system permits data to be collected offline with the Android tablet devices, and to merge with a source data manager laptop computer without the need for Internet connectivity. When Internet connectivity is available to the data manager computer, the system uploads the data to a secure website or server on a cloud-based system. During the pre-testing and training of the COMPASS ACASI/CAPI system, changes to both the translated text and audio-files became necessary. The initial installation of the ACASI program to each Android-tablet device was performed manually from a memory card. The original design for updating the Android-tablets called for manually applying updates to the program and replacements for any audio or graphics using a memory card. In order to streamline and improve the software updating process Population Council added a new module to the ACASI/CAPI program for COMPASS data collections. This new functionality was built using the Microsoft Azure cloud and permitted updates to be made to each Android-device when connected to the Internet rather than requiring updates be made manually (see Fig 2). Thus, an “Update” button was added to each Android tablet which would initiate a connection to the cloud, determine if changes were available, and then retrieve them and update the tablet.
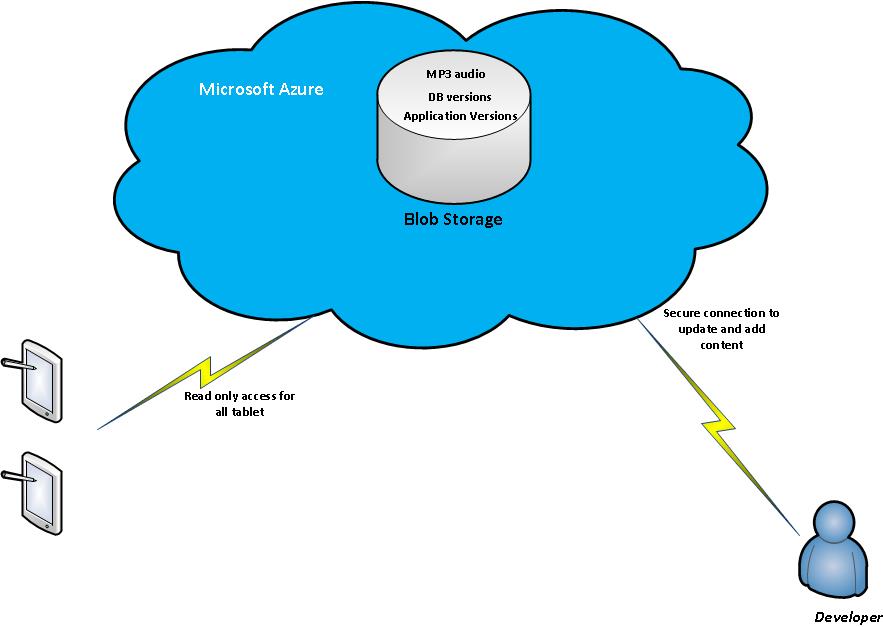


While updates of both visual and audio components of the program were possible throughout data collection as needed, flexibility was required around when updates occurred because of inconsistent internet access. Additional technical issues arose during the course of data collection: for example, in DRC, some of the tablets malfunctioned, and there were cases where enumerators or participants inadvertently deleted the application. The DRC team attempted to remove or lock access to excess applications that came pre-downloaded to the tablet and lock the screen with the questionnaire application to prevent this from reoccurring. Other issues were possible to resolve with remote support from New York.

## Merging data via USB

Each country had one computer with a data manager system installed on it. Data uploading was conducted through a wired USB connection, and did not require the internet. Exported data was then uploaded to a secure cloud-based file when internet was available. In Ethiopia, uploading of data was possible daily, as all data collectors were based in one site and came back to a central location every evening with internet access. In DRC, there were four teams of data collectors working in different locations throughout the week, who returned to the central office once a week. The field teams in DRC adopted a system which allowed them to back-up to a ‘master tablet’ using blue-tooth, as a safeguard against loss or theft during the working week. Originally data was to be merged by using a micro-SD card in each tablet device, transferring the data to the micro-SD card and then inserting the card into the SD card slot on the source data manager computer. However, this was changed to the USB cable that tethered the tablet to the data manager thereby eliminating issues and data loss that can occur when frequently removing and re-inserting the micro-SD cards. In the case of COMPASS, data on cards were used as a back-up if other potential losses of data occurred while out in the field.

## Exporting data routinely for real-time analysis

As with many data collection efforts, proper monitoring of the resulting data is helpful to spot unforeseen trends or issues. One requirement of the COMPASS effort was that all data from each data manager should be able to be exported into simple tabular or matrix formats so that investigators of the team could begin to do analysis. This provided near real-time access to the data despite spotty, slow, or interrupted Internet access. No server was required to do this. The data export was made available via a custom function on the data manager unit which proved to be valuable. In addition, the ability to quickly view what data was collected on each Android tablet device was available so that interviewers could quickly see all the surveys that were collected. This did not include the actual survey responses, but the ID, date, named survey and survey completion status.
